# Study of metastatic kinetics in metastatic melanoma treated with B-RAF inhibitors: Introducing mathematical modelling of kinetics into the therapeutic decision

**DOI:** 10.1371/journal.pone.0176080

**Published:** 2017-05-04

**Authors:** Niklas Hartung, Cécilia T.-K. Huynh, Caroline Gaudy-Marqueste, Antonin Flavian, Nausicaa Malissen, Marie-Aleth Richard-Lallemand, Florence Hubert, Jean-Jacques Grob

**Affiliations:** 1 Department of Clinical Pharmacy and Biochemistry, Institute of Pharmacy, Freie Universität Berlin, Berlin, Germany; 2 Aix-Marseille Université, APHM, Dermatology and skin cancers Department, Marseille, France; 3 Aix-Marseille Université, UMR_S 911 CRO2, Marseille, France; 4 APHM, Hopital Timone, Radiology department, Marseille, France; 5 Aix-Marseille Université, I2M, UMR 7373, CNRS, Centrale Marseille, Marseille, France; Rutgers University, UNITED STATES

## Abstract

**Background:**

Evolution of metastatic melanoma (MM) under B-RAF inhibitors (BRAFi) is unpredictable, but anticipation is crucial for therapeutic decision. Kinetics changes in metastatic growth are driven by molecular and immune events, and thus we hypothesized that they convey relevant information for decision making.

**Patients and methods:**

We used a retrospective cohort of 37 MM patients treated by BRAFi only with at least 2 close CT-scans available before BRAFi, as a model to study kinetics of metastatic growth before, under and after BRAFi. All metastases (mets) were individually measured at each CT-scan. From these measurements, different measures of growth kinetics of each met and total tumor volume were computed at different time points. A historical cohort permitted to build a reference model for the expected spontaneous disease kinetics without BRAFi. All variables were included in Cox and multistate regression models for survival, to select best candidates for predicting overall survival.

**Results:**

Before starting BRAFi, fast kinetics and moreover a wide range of kinetics (fast and slow growing mets in a same patient) were pejorative markers. At the first assessment after BRAFi introduction, high heterogeneity of kinetics predicted short survival, and added independent information over RECIST progression in multivariate analysis. Metastatic growth rates after BRAFi discontinuation was usually not faster than before BRAFi introduction, but they were often more heterogeneous than before.

**Conclusions:**

Monitoring kinetics of different mets before and under BRAFi by repeated CT-scan provides information for predictive mathematical modelling. Disease kinetics deserves more interest

## Introduction

The course of a metastatic melanoma (MM) is currently unpredictable since aggressiveness depends on a network of variables related to tumour and host reaction [1, 2]. B-RAF inhibitors (BRAFi), and MEKi, have led to a major improvement on survival in B-RAF mutated patients [3–7]. However, resistance mechanisms are mostly unpredictable [8–10] and heterogeneity of resistance mechanisms within the same individual [11–14] makes the monitoring difficult, even if liquid biopsies are under development [15]. In addition, resistance may not only be due to molecular events and immune changes may interfere [16].

Growth kinetics, measuring the change in tumor load over time, may be one of the best ways to characterize disease scenarios for therapeutic trials [17]. In a historical cohort of MM patients treated before the era of new treatments, we have shown that initial kinetics of metastases (mets) measured by 2 successive CT-scans is highly predictive for survival [18]. It has also been shown that the homogeneity of response under BRAFi had a prognostic impact on survival [19].

We hypothesized that kinetics of changes in tumor load before and under BRAFi was *per se* reflecting molecular, genetic, and immune mechanisms driving the disease, and could be easier to monitor than a huge number of biomarkers.

Our objective was to show that monitoring of metastatic disease kinetics under targeted therapy is a source of relevant predictive information, which mathematical modelling could use to anticipate events for decision-making.

## Materials and methods

### Study populations

#### BRAFi treated population

To have the simplest approach for modelling, we retrospectively selected from the cohort of MM patients treated in our department (Dermatology and skin cancer department, La Timone Hospital Marseille, France), a series of patients with the following inclusion criteria: stage IIIC or IV AJCC [20], BRAF V600E/K mutation, treatment with BRAFi monotherapy only, and at least two whole-body CT-scans available before BRAFi treatment and at least one CT-scan after BRAFi treatment, all performed on the same machine (in one of the radiology department of our institution), with the same procedure, at most three months apart. Data collection was performed between June 2014 and April 2015.

#### Historical cohort

To estimate the natural kinetics of the metastatic disease, we used a historical cohort of patients who never received any treatment with a demonstrated impact on survival [18] retrospectively selected with the following inclusion criteria: stage IV MM patients treated in our Institution between September 2007 and October 2011 who had two total body computed tomography (CT) scans on the same machine with the same procedure within a maximum of 4 months period after first distant metastases diagnosis, and who meanwhile received either no treatment or only monochemotherapy with dacarbazine or fotemustine or vaccines. Data collection was performed between June and September 2012.

### Assessment of metastatic volumes and kinetics

**Fig 1:**
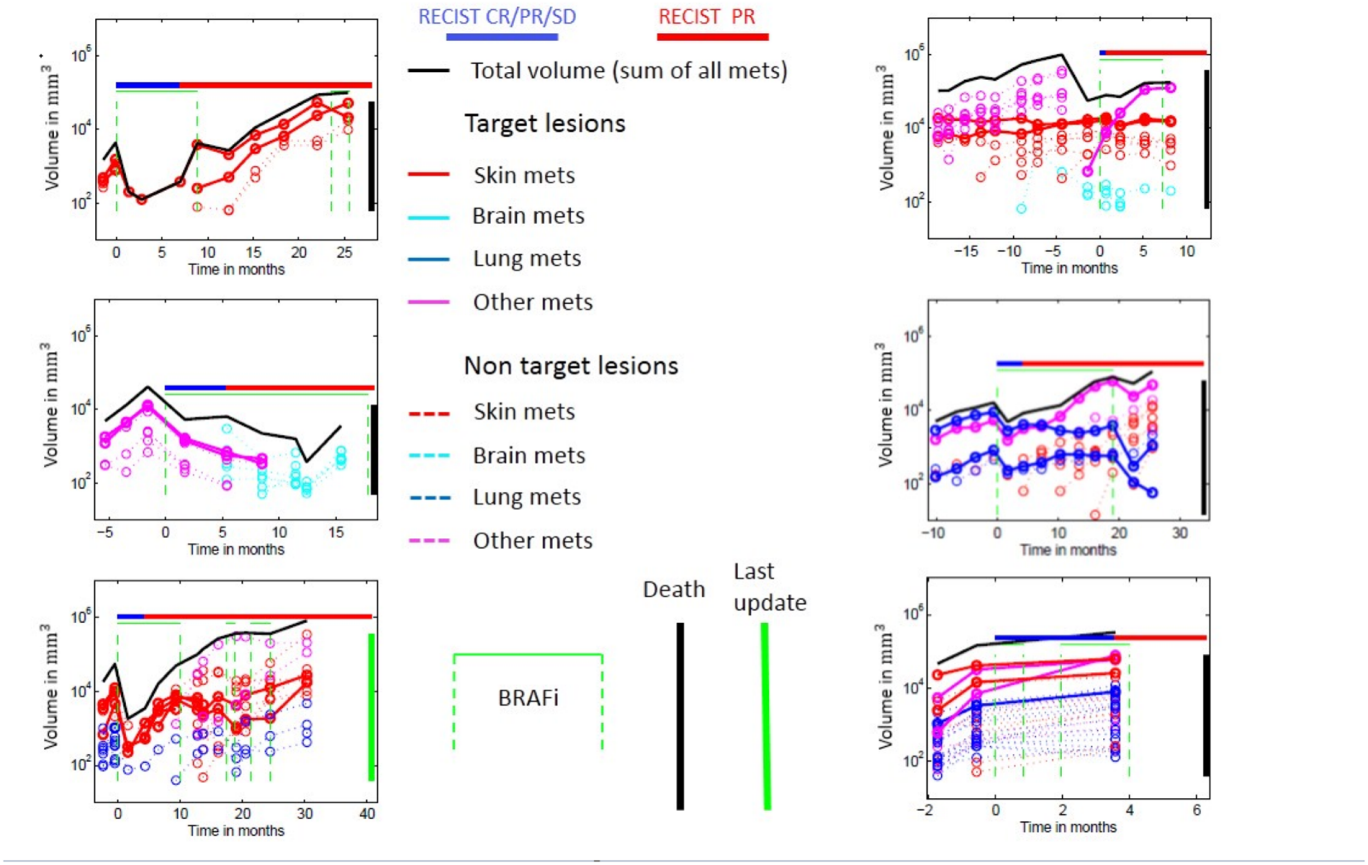
Example plots of metastases volume assessment along the course of the disease in 6 patients.

Volumes of mets were computed using the two native axial measurements and the third measurement from a coronal reconstruction (General Electric Medical Systems, Advantage Workstation 4·4), and assuming an ellipsoidal shape. Each met of which any diameter exceeded 1cm at some time during the follow-up was measured (Fig 1) in all patients at all CT-scans along the follow-up. In this pilot study, measurements were manual and required many hours per patient, which led to limit the sample of patients.

Different variables characterizing metastatic growth kinetics were computed and tested as prognostic markers for survival. Growth kinetics were evaluated as exponential growth rate, linear slope, and relative volume change (RVC). As a measure of the overall kinetic tendency in an individual, kinetics of the total tumor volume and mean kinetics of all mets were calculated. In addition, range and standard deviation of individual metastatic growth kinetics were computed as a measure of homogeneity vs heterogeneity of kinetics between different mets of a given individual. Tumor size-based kinetic indices were computed before and after start of BRAFi therapy (Table 1).

**Table 1:** Tumor size-related measures used in the regression models.

Measure	Timeline		Variable	Formula / symbols
**Tumor burden**	**BL**		Global volume	*V*_*BL*_
			Number of metastases	
**Kinetics**	**BL**	*V*_1_ = *V*_*BBL*_	Exponential growth rate (global / mean)	$a_{Exp}=\frac{\text{log}\left(V_{2}\right)-\text{log}\left(V_{1}\right)}{\Delta t}$
		*V*_2_ = *V*_*BL*_		
	and		Linear slope (global / mean)	$a_{Lin}=\frac{{\text{V}}_{2}-{\text{V}}_{1}}{\Delta t}$
	**IR**	*V*_1_ = *V*_*BL*_	Relative volume change (global / mean)	$\text{RVC}=\frac{{\text{V}}_{2}-{\text{V}}_{1}}{V_{1}}$
		*V*_2_ = *V*_*IR*_		
	**BL**		Lethal burden risk score	$\text{LB}=\frac{a_{\text{Exp}}^{\text{Global}}}{\text{log}\left(V_{\text{lethal}}\right)-\text{log}\left(V_{BL}\right)}$
**Spread of kinetics**	**BL / IR**		Exponential growth rate (SD / range)	
			Linear slope (SD / range)	
			Relative volume change (SD / range)	
**Change in kinetics**	**From BL to IR**		Change in exponential growth rate (global / mean)	
			Change in linear slope (global / mean)	

Abbreviations: BL = baseline, observation prior to BRAFi; BBL = before baseline, observation prior to baseline observation; IR = initial response, first observation after baseline; SD = standard deviation.

All kinetic measures were computed both on the global volume and as a mean measure on the individual metastases. Spread of kinetics was evaluated as standard deviation and range of measures computed on the individual metastases, thus representing a measure of inter-metastasis heterogeneity.

### Modelling the natural spontaneous course of the disease

We used the historical cohort to predict, from initial kinetics, what would have been the natural course of the metastatic disease. An exponential model based on pre-treatment tumor volumes was used to calculate a theoretical total metastatic volume at a time t after first kinetics measure: $V\left(t\right)=V_{BL}\text{exp}\left(a_{\text{Exp}}^{\text{Global}}t\right)$. We assumed that a patient would die when *V*(*t*) reached a critical metastatic burden *V*_lethal_, the most realistic value of which was computed from the historical cohort. The best variable to predict spontaneous survival was found to be the inverse of the time to reach the critical metastatic burden $\frac{a_{\text{Exp}}^{\text{Global}}}{\text{log}\left(V_{\text{lethal}}\right)-\text{log}\left(V_{BL}\right)}$, thereafter called “lethal burden risk score”. More complex tumor growth models were not considered because the risk score had to be derived from only two volume measurements per patient.

### Assessing the predictive value of kinetics indices

Kinetics variables were included in multivariate models with best recognized pejorative predictive markers in patients treated by BRAFi, i.e. RECIST (Response Evaluation Criteria In Solid Tumors version 1.1) progression [21] appearance of new mets under BRAFi, and mixed *vs* uniform initial response [19]. Since RECIST evaluation was not always available, the baseline lesions with largest volumes were considered as theoretical RECIST targets, up to a maximum of five in total and two per organ. A doubling of any non-target lesion volume was considered an unequivocal progression.

### Statistical analyses

Overall survival from start of BRAFi therapy was taken as the clinical endpoint. Patient survival was censored on 16/02/2015 and left-truncated at the time where inclusion criteria were met, i.e. at the first evaluation after start of BRAFi. Impact of covariates available at entering the group at-risk on patient survival was evaluated in Cox proportional hazards (PH) models. To assess whether the tested kinetic indices are independent prognostic markers with respect to the above-mentioned other clinical criteria, they were also evaluated together in multiple explanatory covariate Cox PH models. Furthermore, to account for the appearance of brain metastases and change in RECIST status, multistate models with transition-specific covariates were used [22].

All regression analyses were carried out in R software version 3·1·3 [23] using packages “survival” for Cox and multistate regression [24, 25], and “mstate” for multistate modelling pre and postprocessing [26–28]. Continuous covariates ranging over several orders of magnitude were log-transformed prior to inclusion. The adequacy of functional form of a covariate was evaluated graphically via martingale residuals vs. covariate plots [29]. Likelihood ratios tests were used for model selection, and p-values below 0.05 were considered to be statistically significant.

A survival tree was generated using the “rpart” R package version 4·1–9 with default options [30]. In the algorithm, a two-step procedure is used: in the first step, a tree is built up by recursively partitioning patients into binary subgroups, maximizing the relative risk at each node. Then, to avoid overfitting, the tree is pruned back using a cross-validation criterion, a method that mimics prediction of a future patient not used in building the model. The local full likelihood model allows to deal with censored observation times [31].

## Results

### Study populations

#### BRAFi-treated population

37 patients fulfilled the inclusion criteria. 27 were treated by vemurafenib only, 5 by dabrafenib and 5 successively by vemurafenib and dabrafenib. Population characteristics were as follows: 20 m/17 f, median age 54 (range 20–84 years), AJCC III C in 1 patient, IV M1a in 3 patients, IV M1b in 11 patients and IV M1C in 22 patients. At the end of follow-up, 12 patients were alive on BRAFi, 4 alive with another treatment after BRAFi, 7 died after BRAFi discontinuation, and 14 died during BRAFi therapy.

#### Historical cohort

109 patients of the historical cohort fulfilled the inclusion criteria. Population characteristics are described elsewhere [18] but were similar as in the BRAFi-treated population. In the above-mentioned “lethal burden risk score”, the optimal critical value *V*_lethal_ = 1200 cm3 was determined from the historical cohort.

### Cox regression

A high “lethal burden risk score”, which was the most significant predictors of overall survival (OS) in the historical cohort (p<0·0001) was shown to be also predictor of a poor prognosis in the BRAFi cohort, although less strongly associated (p = 0·04), confirming that initial kinetics before BRAFi is influencing survival under BRAFi. However, the hazard ratio (HR) of a unit increase in risk score tends to belower in the BRAFi cohort, than in the historical cohort with no active treatment: HR = 1·6 (95% CI 1·0–2·3) vs 2·5 (95% CI 1·9–3·4, respectively (p = 0·07 in a test for same HR in both cohorts).

Heterogeneity of initial kinetics expressed as range of RVC before BRAFi, as well as heterogeneity of kinetics in response to BRAFi were significantly predicting poor survival (p = 0·02, and p = 0·005, respectively). Standard deviations were not considered further to describe heterogeneity, due to the high correlation with range of RVC.

As expected, RECIST progressive disease (PD), appearance of new lesions, and a “mixed response” [19] at 1^st^ disease assessment after BRAFi were significantly associated with a poorer outcome (RECIST PD p = 0·004, new lesions p = 0·01, mixed response p = 0·005). Since these three criteria were highly correlated, only RECIST PD, the most significant variable, was retained for further evaluation.

All predictors retained in single explanatory covariate Cox regression, namely “lethal burden risk score”, range of RVC at baseline, and range of RVC at initial response were tested together with RECIST at 1^st^ treatment assessment in a multiple explanatory covariate Cox model. Only range of RVC, hereafter referred to as “response heterogeneity” significantly contributed to the predictive power of the model (p = 0·05). High response heterogeneity was a predictor of poor outcome among RECIST progressors, but not significantly linked to survival in RECIST responders.

### Multistate modelling

**Fig 2:**
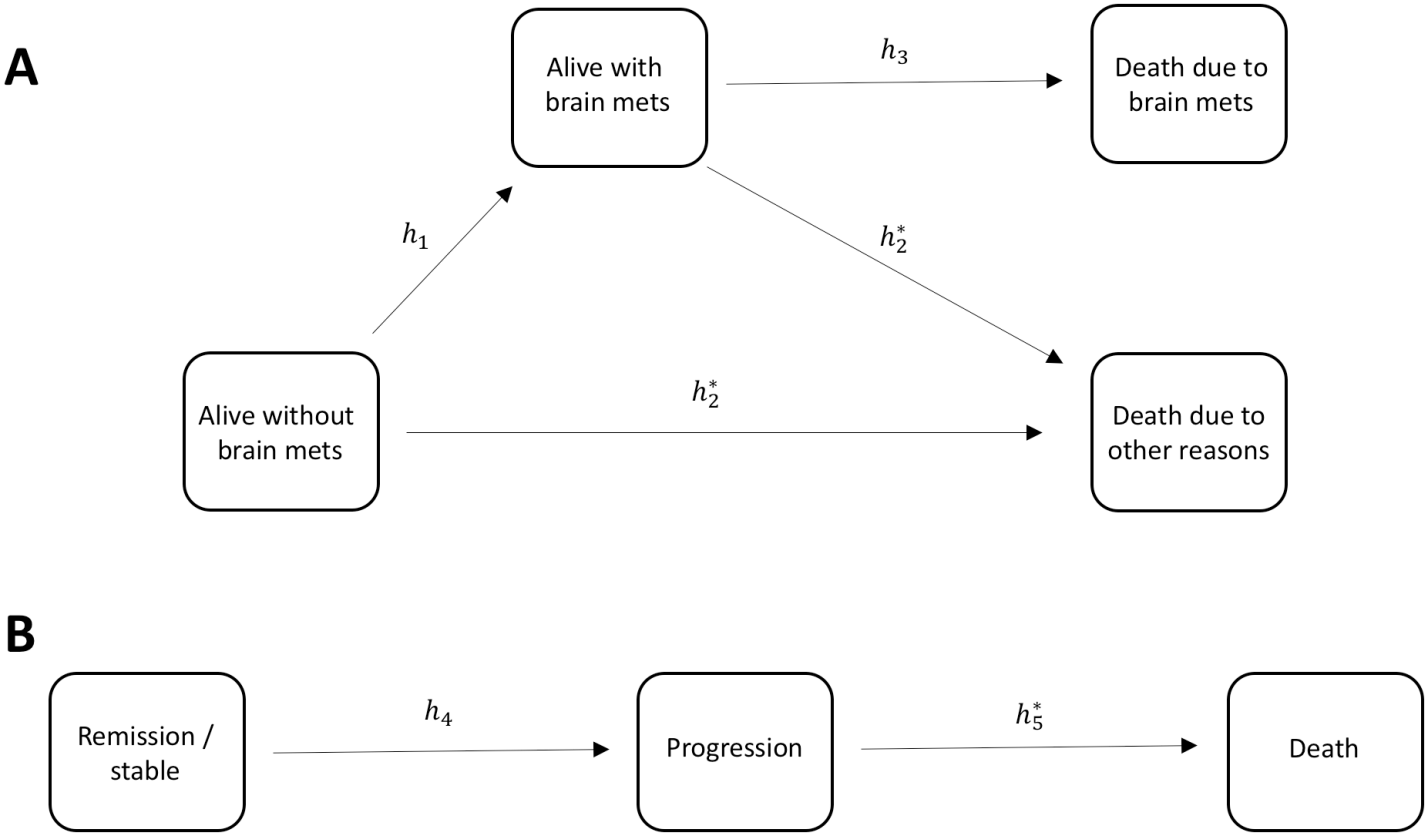
Graphical representation of the developed multistate models. hx: Transition hazards. Asterisks (*) indicate transitions on which covariates are modelled.

To evaluate the impact of dynamic changes of the disease status likely to have an impact on survival, two multistate models were considered, one for brain mets and one for RECIST progression (see Fig 2). Brain-metastatic status was defined by presence or absence of brain mets and by distinguishing the cause of death (linked to or independent from brain mets). Both RECIST PD at 1^st^ assessment and response heterogeneity were associated to the risk of dying for other reasons than brain mets, but not to the risk of developing brain mets, or the risk of dying from brain mets.

Compared to the standard Cox model, the level of significance of “response heterogeneity” increased both independently of RECIST PD at 1^st^ assessment (p = 0·001 in multistate *vs* p = 0·005 in Cox model) and also when considering RECIST PD and response heterogeneity jointly (p = 0·03 in multistate *vs* p = 0·05 in Cox model). A proportional baseline hazard assumption for the different causes of death was tested but not supported by the data [26].

We tested whether response heterogeneity could be also prognostic at a RECIST PD occurring later in the course of the diseases. Considering a multistate model for RECIST progression, response heterogeneity was shown to be a prognostic marker for survival from RECIST PD (p = 0·02), but no link was found between time to RECIST PD and survival from RECIST PD.

### Comparing metastatic kinetics after BRAFi with pre-treatment kinetics

In the 11 patients with CT-scans performed after BRAFi discontinuation, empirical exponential growth rates computed from two volume measurements just before treatment start and two measurements just after treatment were compared to assess the impact of BRAFi on metastatic growth kinetics. Exponential rates of global burden and mean exponential rates were decreased in 9 and 7 patients, stable in 0 and 2, increased in 2 and 2, respectively. Heterogeneity of kinetics between the different mets of a given individual (range of individual growth rates for each met), was decreased in 5 patients, stable in 2, but increased in 5.

## Discussion

Although manual measurement is extremely time-consuming until automatic measures are developed, this study is the proof of concept that a mathematical modelling using kinetics data could be helpful in predicting disease course under BRAFi. Several variables seem important: those characterizing initial kinetics, but more importantly those characterizing chaos in kinetics either before BRAFi or at 1^st^ assessment under BRAFi.

Initial kinetics of MM disease are known to be a good reflect of tumor aggressiveness [18], which can be measured by 2 CT-scans at least 1 month interval before treatment. Ethical considerations about potential loss of time during this interval are not a real issue since usual delay before treatment (eg, validation of mutational status, surgery, pretreatment assessment…) is often longer than a month. A variable like “Lethal burden risk score” representing the predicted spontaneous survival according to initial kinetics, confirms that a high initial kinetics is associated with poor prognosis even in patients treated by BRAFi. This score however shows a trend for a lower impact on the outcome in BRAFi treated patients than in the patients of the historical cohort, suggesting that BRAFi can change the natural history of the disease. It should be noticed that due to the limited cohort size no distinction was made between the two different BRAFi molecules which seem to have similar response profile and response rate.

This study underlines the major prognostic role of heterogeneity of the kinetics as expressed by range of RVC or other similar variables. It is true for heterogeneity of initial kinetics, which may reflect the diversity of molecular pathways at work before treatment, thus increasing the probability of primary resistance in some mets. It is also true for heterogeneity of kinetics at 1^st^ response under BRAFi, which may reflect the diversity of secondary resistance mechanisms in different mets. This evaluation of heterogeneity based on a comprehensive assessment of all mets is supporting the results of a previous study measuring changes in a sample of mets showing that a “mixed” initial response with BRAFi was associated to poorer outcome than a “uniform” response [19]. High heterogeneity in the kinetics of response under BRAFi was still able to add an independent pejorative information to RECIST progression (Cox and multistate models), although initial RECIST progression was one of the most powerful predictor of short survival for any treatment of MM. Furthermore, if we focus on the prediction of survival after RECIST progression, response heterogeneity between mets at progression was a better pejorative marker than a short time to RECIST progression. Unexpectedly, deaths due to brain a mets did not correlate with response heterogeneity perhaps because brain lesions were treated by Gamma-knife in most of our patients.

It is a common perception among clinicians that, when a patient is escaping the B-RAF blockade, the disease is even faster than before treatment. Although potentially biased since cases with early deaths did not permit post-treatment evaluation, our data are not supporting this perception. However, they show more heterogeneity in kinetics after treatment than before, which may be accounted by the fact that BRAFi may promote different resistance mechanisms in the different mets. Describing the whole time course of metastatic growth under BRAFi with a mathematical model could potentially alleviate this bias, but was beyond the scope of this manuscript.

**Fig 3:**
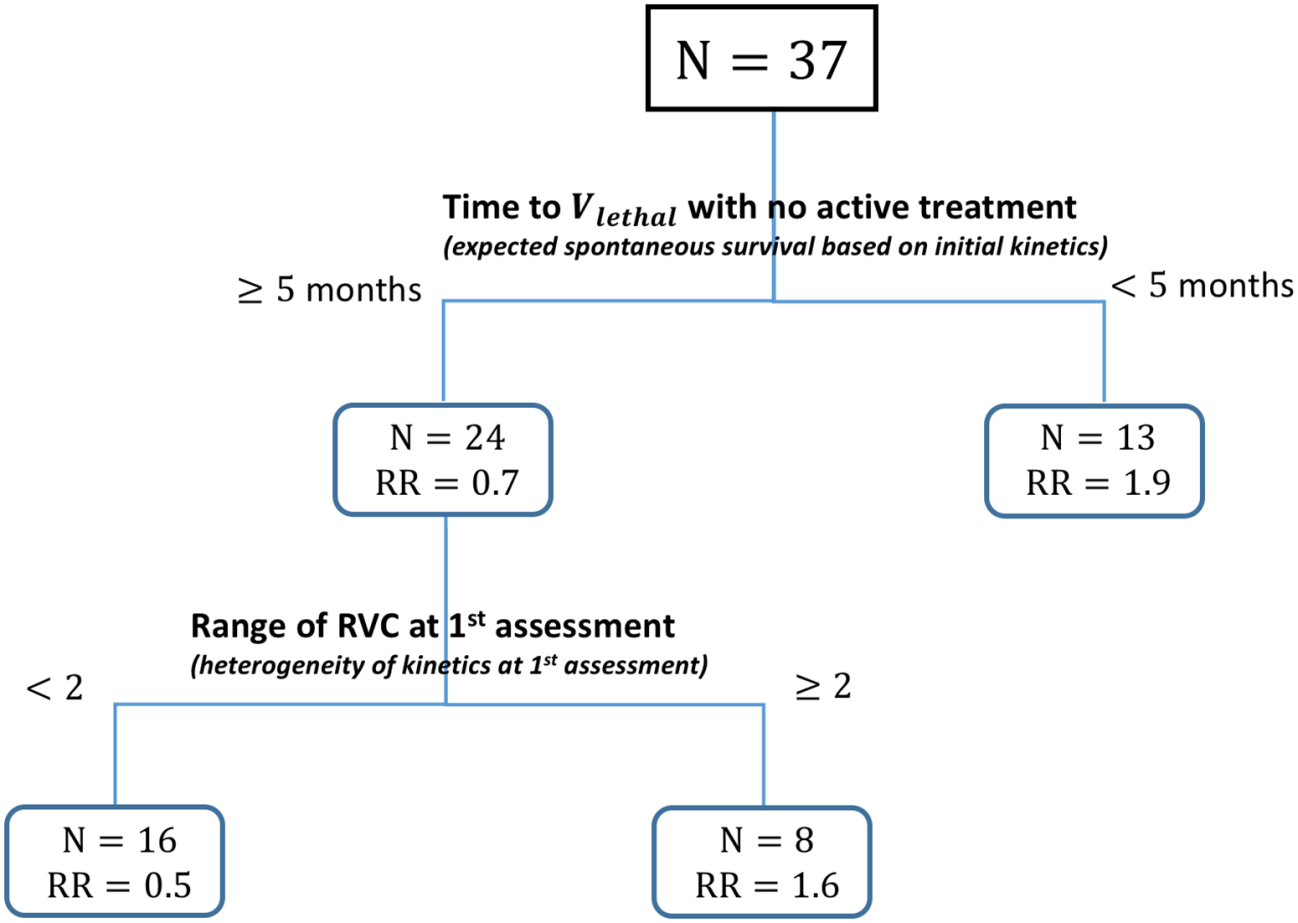
Regression tree for relative risk (RR) of death. Using a partitioning algorithm, 2 kinetics-related factors were selected from the four variables considered in the multiple explanatory covariate Cox model described in the Results. The derived classification algorithm first uses the expected survival deduced from initial kinetics and then heterogeneity of kinetics at 1^st^ assessment expressed as range of RVC.

Monitoring of kinetics can be used as a predictor at 2 levels. As a simple clinical indicator, it is more or less supporting the instinct of the clinician experienced with BRAFi: 1- fast metastatic growth before treatment, especially if there is a clear disorder in growth among the different mets is probably not an ideal situation for BRAFi, and 2-a response to BRAFi with contrasting kinetics in different mets should prompt clinician to switch treatment early if possible. Another level of application is to build up a real predictive model to facilitate therapeutic decision. An example of survival tree has selected two factors (Fig 3): expected spontaneous survival deduced from initial kinetics before BRAFi and 2) heterogeneity of kinetics at 1^st^ assessment of response under BRAFi expressed as range of RVC. This model requires a comprehensive measure of all mets. Such a model could obviously be improved if it was generated from a larger sample of patients, and validated in another sample. This is out of reach for manual measurements but automatization or computer assistance seems quite feasible.

This pilot study shows that kinetics monitoring is a useful source of information in MM patients treated with BRAFi, which may also apply to BRAFi-MEKi combinations. Although most of research is focusing on molecular and immunological biomarkers for the selection and adaptation of the treatment in a given patient, mathematical modelling based on usual imaging systems deserves more interest.

## Supporting information

S1 File Dataset specification. Specification of the variables in Supporting Datasets S1 and S2 Datasets.
(PDF)

S1 Dataset Historical cohort data. (CSV)

S2 Dataset BRAF cohort data. (CSV)
