# Supplementary material for: Study of metastatic kinetics in metastatic melanoma treated with B-RAF inhibitors: Introducing mathematical modelling of kinetics into the therapeutic decision
Source: PLoS One. 2017 May 4;12(5):e0176080. doi: 10.1371/journal.pone.0176080 (PMC5417482; doi:10.1371/journal.pone.0176080)
Supplement: S1 File — Specification of the variables in Supporting Datasets S1 and S2 Datasets. (PDF) [file pone.0176080.s001.pdf]

Dataset specification

Historical cohort:

- ID, patient identifier
- Y0, first measurement after entry into stage IV as sum of volumes of all metastases, computed from whole-body CT scans
- Y1, sum of metastatic volumes measured between 1 and 3 months after Y0
- TTRUNC, time of left-truncation: time interval between the two measurements Y0 and Y1, in days
- OS, overall survival in days, counting from Y0 as a surrogate of entry time into metastatic phase
- STATUS, censoring status: 0 for censored observation, 1 for event (death)

BRAF cohort:

- ID, patient identifier
- OS, overall survival in days, counting from start of first-line BRAFi
- STATUS, censoring status: 0 for censored observation, 1 for event (death)
- TTRUNC, time of left-truncation: time interval between the baseline measurement (directly before start of BRAF treatment) and first evaluation under BRAF, in days
- firstDrug, first BRAFi used: 1 for vemurafenib, 2 for dabrafenib
- logLethalBurdenScore, log-transformed lethal burden score described in the manuscript
- logRangeBL, log-transformed range of relative volume change from the observation before baseline to baseline
- logRangeIR, log-transformed range of relative volume change from baseline to first evaluation under BRAFi
- numberBaselineLesions, number of metastases at baseline
- baselineBurden, sum of volumes of all metastases visible at baseline, in $mm^3$
- uniformMixed, 0: mixed response, 1: uniform response (Menzies et al, PLoS ONE, 2014)
- newAtIR, 0: no new metastases at initial response, 1: new metastases at initial response
- RecistPDatIR, 0: no RECIST PD at initial response, 1: RECIST PD at initial response
- timeRecistProgression, time of RECIST PD or censoring time, in days
- hasRecistProgression, 0: never had RECIST PD, 1: had RECIST PD
- logRangePD, log-transformed range of relative volume change at RECIST progression
- timeFirstBrainMet, time of appearance of first brain metastasis after baseline, in days
- brainMetAtBaseline, 0: no brain metastasis at baseline, 1: at least one brain metastasis at baseline
- causeOfDeath, 0: alive at the end of the study, 1: deach independent of brain metastases, 2: death from brain metastases

All log-transformed covariates are specified in the form log (x+1), where log is the natural logarithm.
